# Phagocytosis of *Aspergillus fumigatus* by Human Bronchial Epithelial Cells Is Mediated by the Arp2/3 Complex and WIPF2

**DOI:** 10.3389/fcimb.2019.00016

**Published:** 2019-02-07

**Authors:** Luka Culibrk, Carys A. Croft, Amreen Toor, S. Jasemine Yang, Gurpreet K. Singhera, Delbert R. Dorscheid, Margo M. Moore, Scott J. Tebbutt

**Affiliations:** ^1^Centre for Heart Lung Innovation, University of British Columbia and St. Paul's Hospital, Vancouver, BC, Canada; ^2^Department of Graduate Studies, Experimental Medicine, University of British Columbia, Vancouver, BC, Canada; ^3^Divison of Respiratory Medicine, Department of Medicine, University of British Columbia, Vancouver, BC, Canada; ^4^Divison of Critical Care Medicine, Department of Medicine, University of British Columbia, Vancouver, BC, Canada; ^5^Department of Biological Sciences, Simon Fraser University, Burnaby, BC, Canada; ^6^Prevention of Organ Failure Centre of Excellence, Vancouver, BC, Canada

**Keywords:** phagocytosis, fungi, aspergillus, cytoskeleton, invasion, host, airway, epithelium

## Abstract

*Aspergillus fumigatus* is an opportunistic fungal pathogen capable of causing severe infection in humans. One of the limitations in our understanding of how *A. fumigatus* causes infection concerns the initial stages of infection, notably the initial interaction between inhaled spores or conidia and the human airway. Using publicly-available datasets, we identified the Arp2/3 complex and the WAS-Interacting Protein Family Member 2 WIPF2 as being potentially responsible for internalization of conidia by airway epithelial cells. Using a cell culture model, we demonstrate that RNAi-mediated knockdown of WIPF2 significantly reduces internalization of conidia into airway epithelial cells. Furthermore, we demonstrate that inhibition of Arp2/3 by a small molecule inhibitor causes similar effects. Using super-resolution fluorescence microscopy, we demonstrate that WIPF2 is transiently localized to the site of bound conidia. Overall, we demonstrate the active role of the Arp2/3 complex and WIPF2 in mediating the internalization of *A. fumigatus* conidia into human airway epithelial cells.

## Introduction

*Aspergillus fumigatus* is a saprophytic filamentous fungus present throughout the world. The spores or conidia of *A. fumigatus* are a potentially infectious agent and are inhaled by most people every day (Latgé, 1999). *A. fumigatus* is known to be capable of behaving as an opportunistic fungal pathogen in immunocompromised individuals, causing a variety of diseases such as allergic bronchopulmonary aspergillosis (ABPA) and invasive aspergillosis (IA). Understanding the mechanisms of interaction between airway epithelial cells (AECs) and the conidia of this organism is vital to develop an understanding of the overall mechanism of infection.

Most infections caused by *A. fumigatus* conidia occur once they have been inhaled by the host, however further knowledge regarding the mechanism of pathogenesis is poorly understood. One hypothesis is that conidia may be internalized by the local airway epithelial cells, whereupon the conidia may germinate and lead to infection (Wasylnka and Moore, 2002; Croft et al., 2016). Specifically, the internalization process occurs via phagocytosis, the process by which cells uptake particulate matter such as pathogens and air pollutants (Gordon, 2016). Since conidia have been demonstrated to survive phagocytosis by non-professional phagocytes and germinate, it is possible that phagocytosis by airway epithelial cells allows them to escape the immune response mediated by macrophages patrolling the airway epithelium. It has been demonstrated that internalization of conidia by airway epithelial cells is dependant on actin polymerization and reorganization, although more detailed mechanistic insights are not yet available (Wasylnka and Moore, 2002; Chen et al., 2015; Toor et al., 2018). One protein complex that is responsible for mediating actin polymerization is the actin reorganization complex 2 and 3 (Arp2/3), which mediates actin reorganization by adding branches to actin filaments (Goley and Welch, 2006). There exist a number of proteins responsible for mediating the activity of Arp2/3, such as Wiskott-Aldrich Syndrome Protein (WASP) and its associated WAS-interacting proteins such as WAS-interacting protein family member 1 and 2 (WIPF1, WIPF2). To address the lack of mechanistic knowledge surrounding the phagocytosis and internalization of *A. fumigatus* conidia, we have employed a data mining approach coupled with *in vitro* cell biology to identify and assess a potential mechanism by which *A. fumigatus* conidia are internalized into airway epithelial cells.

## Methods

Detailed methods have been described in the Supplementary Presentation.

### Data Mining

Statistical analysis was performed using R. Microarray data accessed from the Gene Expression Omnibus was tested for differential expression using the limma package (Smith, 2005). For the RNA-seq data, limma-voom was used (Law et al., 2014). Sparse partial least squares was performed using the spls function from mixOMICS (Lê Cao et al., 2009).

### Culture and Growth Conditions

1HAEo- cells, SV40-transformed normal human airway epithelial cells (Cozens et al., 1992) were routinely grown at 37° C until 80% confluency in Dulbecco's Modified Eagle's Media, 10% fetal bovine serum and 1% Penicillin-Streptomycin. *A. fumigatus* conidia were grown at 30° C as described (Wasylnka and Moore, 2002).

### Immunofluorescence Microscopy

1HAEo- cells were grown to 80% confluency on eight-well chamber slides (Thermo-Fisher). Cells were infected with conidia at a multiplicity of infection of 10 conidia per cell for the indicated periods of time. After infection, samples were washed with PBS, permeabilized with 0.5% Triton X-100 and fixed with 4% paraformaldehyde. Samples were subsequently immunolabeled and prepared for microscopy. Slides were scanned using a Zeiss LSM-880 Inverted Confocal Microscopy with Airyscan technology.

### RNAi of WIPF2

1HAEo- cells were transfected with TriFECTa double stranded dicer-substrate siRNA (DsiRNA) targeting either WIPF2, no known transcript in the human genome (NC-1) (Integrated DNA Technologies) or media as control. Cells were transfected using Lipofectamine RNAiMAX (Thermo-Fisher). Three different siRNA sequences were transfected simultaneously at 3.3 nM concentration each. Transfected cells were grown for 72 h before usage.

### Nystatin Protection Assay

Cells were transfected as described above and infected with *A. fumigatus* conidia for 3 h. After 3 h, unbound conidia were aspirated and media was replaced with media containing nystatin for a further 3 h. Nystatin-containing media was removed and cells were lysed using 0.05% Triton X-100 in water. Lysates were diluted, plated in duplicate onto rich media and colony forming units were counted.

### Arp2/3 Chemical Inhibition and Two-Color Immunofluorescence

Cells were pre-treated with 200 μM CK-666 (Sigma-Aldrich), an Arp2/3 inhibitor in media or equivalent volume of DMSO for 30 min prior to infection with *A. fumigatus* conidia for 3 h under the same treatment conditions. Two-color immunofluorescence was used as described previously (Wasylnka and Moore, 2002) to assess the degree of internalization.

## Results

### Data Mining Identifies Candidate Genes

In order to identify candidate genes for analysis, we first assessed data obtained in a previous experiment analyzing the interaction of *A. fumigatus* conidia and human airway epithelial cells (Gomez et al., 2010; Oosthuizen et al., 2011). We identified 652 and 118 genes as being differentially expressed post-infection in the human and the *A. fumigatus* datasets, respectively (FDR < 0.15) (Supplementary Tables 1, 2). To further filter our dataset, we used sparse partial least squares (sPLS) (Rohart et al., 2017) to identify highly correlated differentially expressed gene pairs between the human and *A. fumigatus* datasets. We selected the top 20 gene pairs from both datasets for further analysis.

We hypothesized that the airway epithelial cells may internalize conidia without regard for their identity. In an attempt to lend strength to this hypothesis, we accessed publicly-available RNA-seq datasets relevant to this interaction. Specifically we selected experiments which used primary airway epithelial cells that were infected *in vitro* by a pathogen for under 8 h. We identified three datasets matching these criteria (Table 1). The data were aligned and analyzed for differential expression post-infection (FDR < 0.15) (Supplementary Table 3). Two genes identified within the RNA-seq dataset were replicated in the sPLS dataset: RNA Pseudouridylate Synthase Domain Containing 4 (*RPUSD4*) and WAS-interacting protein family member 2 (*WIPF2*). We elected to specifically evaluate WIPF2's impact on the internalization of *A. fumigatus* conidia further, as the potential mechanism of action of *WIPF2*, a gene involved in actin polymerization was much more clear than *RPUSD4*, an RNA-binding protein.

**Table 1:** Indicated RNA-seq datasets corresponding to bronchial epithelial cells infected by various pathogens were downloaded from the NCBI SRA.

**SRA Accession**	**Treatment**
SRR1565944	Rhinovirus
SRR1565945	Rhinovirus
SRR1565946	Rhinovirus
SRR1565947	Rhinovirus
SRR1565948	Rhinovirus
SRR1565949	Rhinovirus
SRR1565938	Control
SRR1565939	Control
SRR1565940	Control
SRR1565941	Control
SRR1565942	Control
SRR1565943	Control
SRR1714493	*H. Influenzae*
SRR1714495	*H. Influenzae*
SRR1714481	Control
SRR1714483	Control
SRR1714482	Control
ERR894734	Control
ERR894735	Control
ERR894736	Control
ERR894737	Control
ERR894761	*P. aeruginosa*
ERR894760	*P. aeruginosa*
ERR894759	*P. aeruginosa*
ERR894758	*P. aeruginosa*

*The collected dataset was tested for gene expression changes between infected and uninfected samples*.

### WIPF2 Is Transiently Localized to the Site of *A. fumigatus* Adhesion

**Figure 1:**
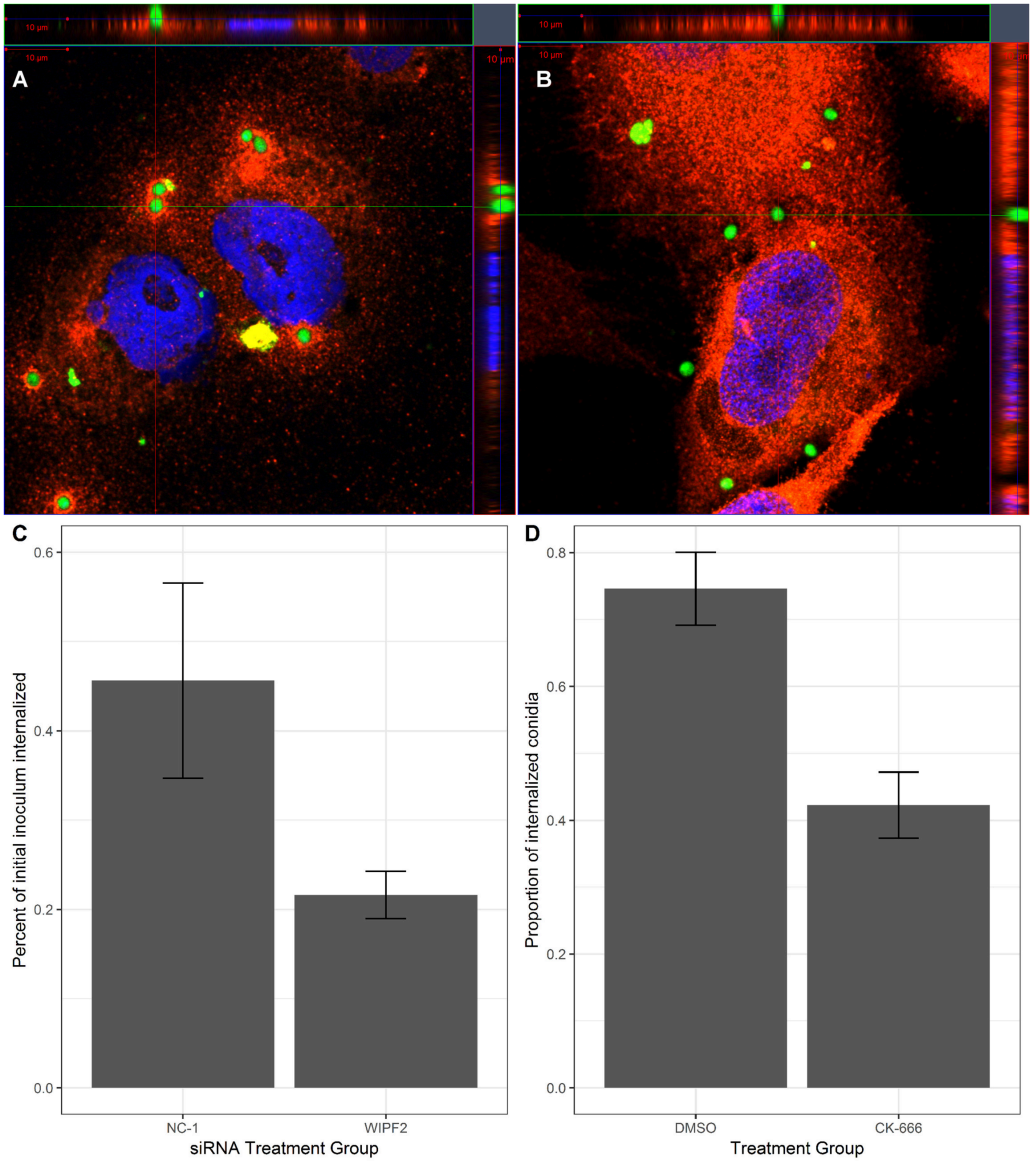
WIPF2 and Arp2/3 mediate internalization of conidia into 1HAEo- cells. **(A,B)** 1HAEo- cells were infected with GFP-expressing *A. fumigatus* conidia for 15 min **(A)** or 1 h **(B)**. After infection, samples were immunolabeled using anti-WIPF2 antibody and DAPI and imaged using super-resolution confocal microscopy. **(C)** 1HAEo-cells were transfected with siRNA targeting no known gene in the genome (NC-1) or WIPF2. The nystatin protection assay was used to assess the extent of internalization. *P* < 0.05 (Student's *T*-test). **(D)** 1HAEo-cells were treated with CK-666, an Arp2/3 inhibitor or DMSO vehicle before infection by GFP-expressing *A. fumigatus* conidia. Extracellular conidia were labeled by immunolabeling without permeabilization followed by manual counting. The proportion of observed conidia that were internalized is shown. *P* < 0.05 (Student's *T*-test).

Previously, actin polymerisation has been demonstrated to occur at the site of adhesion of *A. fumigatus* conidia to AECs (Wasylnka and Moore, 2002). The Arp2/3 complex is known to be responsible for extending actin filaments and mediate the formation of filopodia (Goley and Welch, 2006). WAS-interacting protein family member 2 (*WIPF2*), a gene whose protein is involved in mediating the activity of the Arp2/3 complex via activity of the Wiskott-Aldrich syndrome protein (WASP) (Takenawa and Miki, 2001) was selected as a surrogate for the Arp2/3 pathway in this experiment. 1HAEo- cells were infected with GFP-expressing *A. fumigatus* conidia for 15 or 60 min before immunolabeling with anti-WIPF2 antibody and imaging with super-resolution airyscan microscopy. DAPI was used as a nuclear counter-stain. Localization of WIPF2 fluorescent signal with GFP signal from the conidia was observed to be localized as a “ring” around the conidia 15 min post-infection (Figure 1A). Furthermore, 60 min post-infection the WIPF2 signal was observed to be diffused throughout the cells (Figure 1B). This indicated that WIPF2 was involved in the immediate early stages of interaction between 1HAEo- cells and conidia. Orthographic projection of Z-stacks indicated that the conidia were not internalized at either time point (Figures 1A,B).

### WIPF2 Controls Internalization of Conidia by 1HAEo- Cells

The role of actin polymerization in the interaction of AECs and conidia is the mediation of internalization of the conidia by the AECs (Wasylnka and Moore, 2002; Croft et al., 2016). We hypothesized that knockdown of WIPF2 in AECs would lead to a reduction in internalization rates. We demonstrated effective depletion of WIPF2 protein product in 1HAEo- cells by RNAi 72 h post-transfection using three siRNA targeting WIPF2 (RNAi-WIPF2 cells) compared to non-targeting siRNA (RNAi-NC-1 cells) (Supplementary Figure 1). In order to quantify differential internalization between RNAi-WIPF2 cells and RNAi-NC-1 cells, we used the nystatin protection assay, a previously published method adapted from the gentamicin protection assay (Wasylnka and Moore, 2002; Chen et al., 2015). Briefly, 1HAEo- cells were incubated with conidia for a period of 3 h, after which time the inoculum was aspirated and replaced with media containing nystatin. Nystatin is a fungicide which kills conidia but cannot penetrate the cell membrane. After a further 3 h, the nystatin-containing media was removed and cells were gently lysed to release internalized conidia. Lysates were subsequently diluted and plated onto rich media and colony-forming units were counted to approximate total internalized conidia. We demonstrated that RNAi-WIPF2 cells internalized 52% fewer conidia compared RNAi-NC-1 cells (Figure 1C) (Supplementary Table 4).

### The Arp2/3 Complex Mediates Internalization of Conidia by 1HAEo- Cells

Although we have specifically interrogated the role of WIPF2 on the internalization of conidia, it is an upstream regulator of the actual complex responsible for actin reorganization: Arp2/3. Using CK-666, a small molecule inhibitor of Arp2/3 (Hetrick et al., 2013) we performed small molecule inhibition of Arp2/3 and measured internalization of conidia via fluorescence microscopy. In the cultures treated with CK-666, we observed a 43% reduction in internalization when compared with DMSO vehicle (Figure 1D).

## Discussion

The human Arp2/3 complex is responsible for actin rearrangement and is regulated by a number of proteins. Particularly, WASP and associated WAS-interacting proteins are known to mediate this process. One notable WAS-interacting protein is WIPF2, which binds cooperatively with WASP to mediate actin filopodia formation by Arp2/3 (Takenawa and Miki, 2001). Filopodia, or actin microspikes have been described as the “phagocytic tentacles” of macrophages (Goley and Welch, 2006; Kress et al., 2007). Our data suggest that the internalization of *A. fumigatus* conidia by AECs occurs through this mechanism.

The early stages of interaction between *A. fumigatus* conidia and AECs is characterized by adhesion of conidia and the initiation of actin polymerization (Croft et al., 2016). Our data indicate that WIPF2 may have an activating role in this process, as we observed localization of WIPF2 to conidia only during the earliest stages of this interaction. Since WIPF2 mediates activity of WASP, which in turn mediates activity of Arp2/3, this indicates that WIPF2 may play a regulatory role in activating WASP at the site of adhesion and subsequently dissociating once the process of actin polymerization has been initiated. WIPF2 is predicted to be a highly disordered protein, which are known to have regulatory roles and is consistent with this model (Berman et al., 2000; Oldfield and Dunker, 2014).

WIPF2 plays a key role in mediating the activity of Arp2/3. Despite being two proteins upstream of the actual mediator of actin polymerization, Arp2/3, inhibition of WIPF2 protein expression resulted in a large decrease in the internalization of conidia. This decrease was comparable to the reduction in internalization we observed after directly inhibiting Arp2/3 with a chemical inhibitor, indicating that WIPF2 plays a central and possibly rate-limiting role in this process.

In healthy individuals, conidia are removed from the airway by mucociliary action or are phagocytosed and destroyed by macrophages (Bhatia et al., 2011; Lee et al., 2016). It has been demonstrated that while macrophages are capable of killing conidia after phagocytosis and formation of the phagolysosome, conidia are able to survive and germinate within non-professional phagocytes (Wasylnka and Moore, 2003). Within immunocompromized individuals, macrophages may be less abundant or incompetent, allowing time for internalization of conidia by AECs. This would lead to germination of conidia and potentially development of disease. Further research into the role of internalization of conidia on pathogenesis will be necessary in order to test this hypothesis.

In the present study, we propose a model governing the initial stages of interaction between AECs and conidia. The role of other agents in the Arp2/3 pathway in this dual-organism interaction are unknown. Since WIPF2 was identified in our data screen which was not specific to *A. fumigatus* or even fungi, it is possible that this mechanism is not specific to the internalization of *A. fumigatus* conidia, or even any organism. Further research will be required in a number of areas, such as the kinetics and regulation of this biomechanical process, as well as determination of the generalizability of our model. Overall we provide evidence implicating a specific mechanistic pathway, the Arp2/3 pathway as being responsible for a key process in this host-pathogen system.

## Data Availability Statement

The datasets analyzed for this study can be found in the NCBI SRA (https://www.ncbi.nlm.nih.gov/sra). Dual-organism microarray data may be found in the NCBI Gene Expression Omnibus (https://www.ncbi.nlm.nih.gov/geo) under accession GSE16637.

## Author Contributions

LC: performed experiments and wrote the manuscript; CC, GS, SY, and AT: provided substantial contributions to study design and acquisition of data; MM, DD, and ST: provided substantial contributions to study design. All authors contributed in the revision of the manuscript and approve of submission.

### Conflict of Interest Statement

The authors declare that the research was conducted in the absence of any commercial or financial relationships that could be construed as a potential conflict of interest.

## Supplementary Material

Supplementary Table 1 Human genes differentially expressed in AECs after *A. fumigatus* exposure. Microarray data were accessed from the gene expression omnibus and analyzed for differential expression (FDR < 0.15).

Supplementary Table 2 *A. fumigatus* genes differentially expressed after co-incubation with AECs. Microarray data were accessed from the gene expression omnibus and analyzed for differential expression (FDR < 0.15).

Supplementary Table 3 Human genes differentially expressed in primary AECs after infection with a variety of pathogens. RNA-seq data were accessed from the NCBI SRA and processed for differential expression (FDR < 0.15).

Supplementary Table 4 Raw counts of *A. fumigatus* colonies after nystatin protection assay.
