# Supplementary material for: Phagocytosis of Aspergillus fumigatus by Human Bronchial Epithelial Cells Is Mediated by the Arp2/3 Complex and WIPF2
Source: Front Cell Infect Microbiol. 2019 Feb 7;9:16. doi: 10.3389/fcimb.2019.00016 (PMC6375057; doi:10.3389/fcimb.2019.00016)
Supplement: Supplementary file 5 [file Presentation_1.pdf]

Supplementary Material:
Phagocytosis of *Aspergillus fumigatus* by human bronchial epithelial cells is mediated by the Arp2/3 complex and *WIPF2*

1 SUPPLEMENTARY DATA

Supplementary Figures

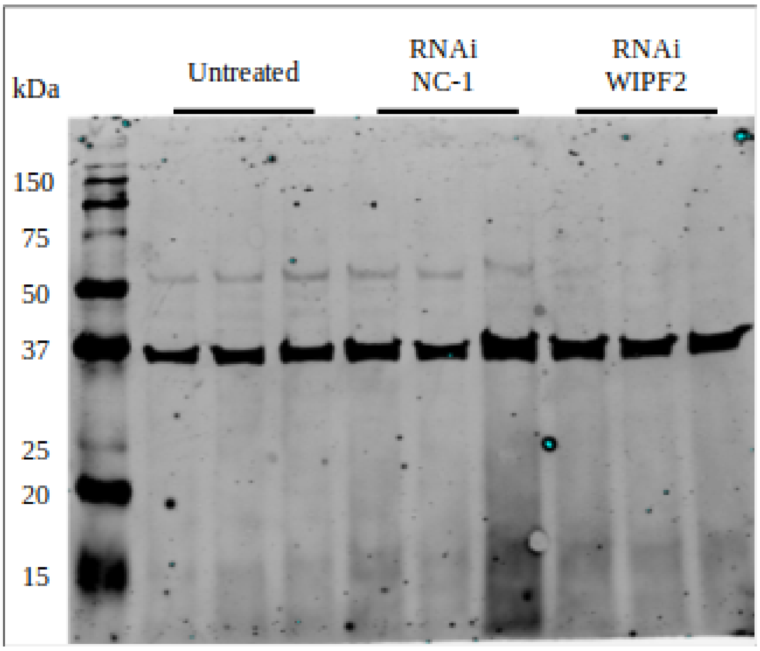

Figure S1. 1HAEo- cells were transfected for 72 hours with siRNA targeting WIPF2 (RNAi WIPF2), no known target in the human genome (RNAi NC-1) or no siRNA as control (Untreated). Cells were then lysed and protein lysates used in SDS-PAGE followed by Western Blot. Blots were stained for WIPF2 (54kDa) and GAPDH as a loading control (37 kDa).

Supplementary Methods

A. fumigatus culture and growth conditions

GFP-transformed ATCC13073 A. fumigatus conidia were prepared as described, with two modifications (Wasylnka and Moore, 2002). The conidia were grown at 30°C and were grown on yeast extract-agar-glucose media.

Cell culture and growth conditions

1HAEo- cells, SV40-transformed normal human airway epithelial cells (Cozens et al., 1992) were grown at 37°C with 5% CO2 in in Dulbecco's Modified Eagle Medium (DMEM; Thermo Fisher Scientific,

12491015) supplemented with 10% fetal bovine serum (FBS; Thermo Fisher Scientific, 10082147) and 1% penicillin-streptomycin (Thermo Fisher Scientific, 15140122). Subcultures were routinely performed as cells reached 80% confluence.

RNA interference of WIPF2

Transfections were performed using Lipofectamine RNAiMAX transfection reagent (Thermo Fisher Scientific, 13778100) with the TriFECTa RNAi kit (Integrated DNA Technologies, hs.Ri.WIPF2.13). The TriFECTa RNAi kit contained three species of siRNA that targeted the transcript encoding WIPF2. The kit also contained NC-1 siRNA, which does not target any known sequence in the human or A. fumigatus transcriptome. A 1:50 solution of Lipofectamine RNAiMAX in DMEM was combined with an siRNA solution that contained the three WIPF2 siRNA species in equal molar proportions of 39.6μL in DMEM. After 5 minutes of incubation at room temperature, 100μL of transfection solution was added to each well of a 24-well plate. 1HAEo- cells (30,000) were added to each well in DMEM supplemented with 10% FBS yielding a final volume of 600μL with siRNA concentrations of 3.3 nM each. NC-1 siRNA was transfected with a final concentration of 10 nM. Transfected cells were grown for 72 h until experimental usage.

Chemical Inhibition of Arp2/3

For chemical inhibition experiments, 200μM CK-666 (Sigma-aldrich, SML0006) in DMSO or equivalent volume of DMSO alone in media was added to cells 30 minutes before infection. Cells were infected for three hours as in the Nystatin Protection Assay experiment (above). Two-color immunofluorescence was used to assess internalization of *A. fumigatus*, and was performed as described previously (Wasylnka and Moore, 2002), with five fields of view quantified per sample.

Sodium dodecyl sulfate polyacrylamide gel electrophoresis (SDS-PAGE) and western blot

After washing cells with PBS, they were lysed by scraping in 100μL of lysis buffer 6 (R&D Biosystems, 895561) supplemented with protease inhibitors (Roche, 11697498001). The lysis buffer contained phosphatase inhibitors from the manufacturer. Each lysate (25μL) was collected, combined with 5μL of sample buffer (2% SDS, 10% glycerol, 40mM Tris, 5% β-mercaptoethanol) and denatured for 5 minutes at 90°C. Samples were separated on a 10% 0.75 mm SDS-PAGE gel and proteins were transferred onto a nitrocellulose membrane using semi-dry electrophoretic transfer at 25V for 45 minutes using a Trans-blot Turbo apparatus (Bio-Rad). Membranes were blocked at 4°C overnight in 5% bovine serum albumin (BSA) in PBS containing 0.4% Tween-20 (PBS-0.4T). Membranes were probed for one hour using 1:2000 rabbit anti-GAPDH (Cell signaling, 2118S) and 1:1000 rabbit anti-WIPF2 antibody (Sigma-Aldrich, HPA024467) in PBS-0.4T. Membranes were washed three times for five minutes each in PBS-0.4T and probed with 1:10000 IRDye 680 Red goat anti-rabbit antibody (Li-Cor, 926-68071). Membranes were scanned using a Li-Cor Odyssey infrared scanner.

Nystatin Protection Assay

This assay has been previously validated in studies investigating the internalization of conidia into airway epithelial cells (Wasylnka and Moore, 2002; Gomez et al., 2010; Chen et al., 2015). Briefly, 1HAEo- cells were transfected for 72 h as described above with siRNA targeting WIPF2, NC-1 siRNA, or left untreated as control. Cells were then infected for three hours with 10^6 conidia in DMEM at 37°C. Conidia solutions were then aspirated, cultures were washed once with PBS-T and treated with 25μg/mL Nystatin and 0.5% DMSO in DMEM for three hours at 37°C. Cells were washed three times with PBS-T and lysed for 15

minutes at room temperature with 0.05% Triton X-100 in sterile dH2O. Lysates were diluted with sterile dH2O, plated in duplicate onto YAG and grown for 24 h at 37°C. Colonies were counted manually.

Immunofluorescence of WIPF2

1HAEo- cells were grown as described above in 8-well tissue culture treated chamber slides (Thermo Fisher Scientific, 154534PK). Cells were infected with conidia at a MOI of 10 in DMEM for the indicated periods of time. After infection, cells were washed three times with PBS-T and fixed with 4% paraformaldehyde in PBS for 20 minutes. Cells were washed three times with PBS and permeabilized for five minutes using cytoskeleton solution (10mM MES, 138mM KCl, 3mM MgCl, 2mM EGTA, 320mM sucrose, pH 6.1) supplemented with 0.5% Triton X-100. Cells were washed three times with PBS and blocked for one hour at 4°C in PBS with 5% BSA. Samples were then immunolabeled with 1:200 rabbit anti-WIPF2 antibody in blocking solution for one hour, washed three times with PBS, and incubated with 1:400 goat-anti rabbit Alexa-fluor 596 antibody (Jackson laboratories, 111-585-045) in blocking solution for one hour. Samples were washed three times with PBS and treated with 0.1μg/mL of DAPI for five minutes to stain nuclei. Cells were washed three times with PBS, and cover slipped with Fluoromount medium (Sigma-Aldrich, F4680) after removing the chambers. Finally, the slides were sealed with nail polish.

Airyscan confocal microscopy

Slides were imaged using a Zeiss LSM-880 inverted confocal microscope with Airyscan technology and ZEN black software (Zeiss). The microscope settings were: frame size 1488x1488px, pixel size 0.05μm, pixel dwell 1.41μs with 4x averaging in the left-right direction with the mean method and 12-bit colour depth. For airyscan processing, “auto” was used. Z-stack images were obtained as 11 Z-slices with 0.6μm spacing. Pinhole size/gain for each channel in the 15 minute post-infection sample was 52.5/900R, 42.8/850G, and 35.9/650B (R = red, G = green, B = blue). For the 60-minute sample, the pinhole size and gain were 85.1/900R, 79.3/850G, and 48.0/650B.

REFERENCES

Chen, F., Zhang, C., Jia, X., Wang, S., Wang, J., Chen, Y., et al. (2015). Transcriptome Profiles of Human Lung Epithelial Cells A549 Interacting with Aspergillus fumigatus by RNA-Seq. *PLoS ONE* 10, e0135720. doi:doi:10.1371/journal.pone.0135720

Cozens, A. L., Yezzi, M. J., Yamaya, M., Steiger, D., Wagner, J. A., Garber, S. S., et al. (1992). A transformed human epithelial cell line that retains tight junctions post crisis. *In Vitro Cell Dev Biol* 28A, 735–44

Gomez, P., Hackett, T. L., Moore, M. M., Knight, D. A., and Tebbutt, S. J. (2010). Functional genomics of human bronchial epithelial cells directly interacting with conidia of Aspergillus fumigatus. *BMC Genomics* 11, 358. doi:1471-2164-11-358[pii]\n10.1186/1471-2164-11-358

Wasylnka, J. A. and Moore, M. M. (2002). Uptake of Aspergillus fumigatus conidia by phagocytic and nonphagocytic cells in vitro: Quantitation using strains expressing green fluorescent protein. *Infect Immun* 70, 3156–3163. doi:10.1128/IAI.70.6.3156-3163.2002
